# Dual Targeting of Sorafenib-Resistant HCC-Derived Cancer Stem Cells

**DOI:** 10.3390/curroncol28030200

**Published:** 2021-06-11

**Authors:** Ritu Shrestha, Kim R. Bridle, Lu Cao, Darrell H. G. Crawford, Aparna Jayachandran

**Affiliations:** 1 Faculty of Medicine, The University of Queensland, Brisbane, QLD 4120, Australia; ritu.shrestha@uq.edu.au (R.S.); k.bridle@uq.edu.au (K.R.B.); l.cao1@uq.edu.au (L.C.); d.crawford@uq.edu.au (D.H.G.C.); 2 Gallipoli Medical Research Institute, Greenslopes Private Hospital, Brisbane, QLD 4120, Australia; 3 Fiona Elsey Cancer Research Institute, Ballarat, VIC 3350, Australia

**Keywords:** hepatocellular carcinoma, cancer stem cells, sorafenib, epithelial-to-mesenchymal transition, immune checkpoint

## Abstract

Sorafenib, an oral multi-tyrosine kinase inhibitor, has been the first-line therapy for the treatment of patients with advanced HCC, providing a survival benefit of only three months in approximately 30% of patients. Cancer stem cells (CSCs) are a rare tumour subpopulation with self-renewal and differentiation capabilities, and have been implicated in tumour growth, recurrence and drug resistance. The process of epithelial-to-mesenchymal transition (EMT) contributes to the generation and maintenance of the CSC population, resulting in immune evasion and therapy resistance in several cancers, including HCC. The aim of this study is to target the chemoresistant CSC population in HCC by assessing the effectiveness of a combination treatment approach with Sorafenib, an EMT inhibitor and an immune checkpoint inhibitor (ICI). A stem-cell-conditioned serum-free medium was utilised to enrich the CSC population from the human HCC cell lines Hep3B, PLC/PRF/5 and HepG2. The anchorage independent spheres were characterised for CSC features. The human HCC-derived spheres were assessed for EMT status and expression of immune checkpoint molecules. The effect of combination treatment with SB431542, an EMT inhibitor, and siRNA-mediated knockdown of programmed cell death protein ligand-1 (PD-L1) or CD73 along with Sorafenib on human HCC-derived CSCs was examined with cell viability and apoptosis assays. The three-dimensional spheres enriched from human HCC cell lines demonstrated CSC-like features. The human HCC-derived CSCs also exhibited the EMT phenotype along with the upregulation of immune checkpoint molecules. The combined treatment with SB431542 and siRNA-mediated PD-L1 or CD73 knockdown effectively enhanced the cytotoxicity of Sorafenib against the CSC population compared to Sorafenib alone, as evidenced by the reduced size and proliferation of spheres. Furthermore, the combination treatment of Sorafenib with SB431542 and PD-L1 or CD73 siRNA resulted in an increased proportion of an apoptotic population, as evidenced by flow cytometry analysis. In conclusion, the combined targeting of EMT and immune checkpoint molecules with Sorafenib can effectively target the CSC tumour subpopulation.

## 1. Introduction

Hepatocellular carcinoma (HCC) is a major global health problem with increasing incidence and poor survival rates [1,2]. Curative treatments for HCC patients are only feasible at early stages, with limited treatment modalities for advanced HCC [3,4,5]. The Food and Drug Administration (FDA) approved Sorafenib as the first drug for the treatment of unresectable advanced HCC [6].

The potential survival benefit with Sorafenib is minimal with an extension in the survival time of only three months in patients with HCC [6,7,8]. Furthermore, only one-third of advanced HCC patients respond to Sorafenib treatment due to the development of Sorafenib resistance [6,9,10]. Effective treatment alternatives for patients with HCC resistant to Sorafenib are still lacking. Elucidating the mechanisms of Sorafenib resistance and exploring better treatment strategies to improve therapeutic outcomes in HCC is warranted. Studies have proposed several mechanisms of Sorafenib resistance. Primary or intrinsic resistance to Sorafenib in HCC patients mostly results from genetic heterogeneity, while secondary or acquired resistance to Sorafenib treatment results from several resistance factors [11]. The commonly reported mechanisms attributed to Sorafenib resistance are epithelial-to-mesenchymal transition (EMT) [12,13,14], cancer stem cells (CSCs) [15,16], autophagy [17,18], hypoxia [19,20] and deregulated signal transduction including EGFR activation [21,22], c-Jun activation [23,24,25] and Akt activation [26,27].

EMT, a major reversible cellular process allowing cells to transform from epithelial to motile phenotypes, is well known for its role in embryonic development processes, organ fibrosis and the progression and metastasis of many cancers, including HCC [28,29,30]. The process of EMT has been implicated in tumour cells acquiring resistance to chemo and radiotherapies [28]. Studies have revealed a significant link between EMT and Sorafenib resistance in HCC [28,31,32,33,34]. Similarly, studies have demonstrated that EMT also contributes to the generation and maintenance of the CSC population in cancers, including HCC [32,35,36,37].

CSCs are a tumour-initiating subset of tumour cells with stem-cell-like properties for self-renewal, proliferation, production of heterogeneous progeny, metastasis and chemotherapy resistance [38,39,40]. Hepatic CSCs have been isolated and characterised using multiple cell surface markers, including CD133, CD44, EpCAM, CD90, CD13 and others [38,41]. Despite accounting for only a small portion of a tumour, CSCs are a chemoresistant subpopulation contributing to therapy failure in most cancers, including HCC [42,43].

Upon undergoing EMT, cells are reported to possess CSC-like features and vice versa as a result of crosstalk between EMT and CSCs [39]. Studies have demonstrated that EMT is activated in Sorafenib-resistant cells along with an enrichment of the CSCs [12,44]. However, specific studies on the modulation of EMT, CSC and Sorafenib resistance in HCC are still lacking.

Recently, the FDA has approved immune checkpoint inhibitors (ICIs), Nivolumab and Pembrolizumab, against programmed cell death protein-1 (PD-1), for treatment of HCC patients previously treated with Sorafenib [45,46]. Furthermore, several combination approaches with ICIs, such as Nivolumab combined with Ipilimumab (anti-cytotoxic T-lymphocyte-associated protein-4 (CTLA-4)) and Atezolizumab (anti-PD-L1) combined with Bevacizumab, have been approved to improve therapeutic efficacy in HCC patients [47,48]. CSCs and EMT have been closely linked with the development of immune evasion in cancers [49,50,51]. However, little is known about the Sorafenib–EMT–CSC–ICI axis.

In this study, we utilised a sphere-forming assay to enrich CSCs from human HCC cell lines based on the initial protocol established by Reynolds and Weiss to generate neurospheres from adult brain [52]. The sphere-forming assay for CSC enrichment uses a serum-free medium supplemented with appropriate mitogens such as epidermal growth factor (EGF), basic fibroblast growth factor (bFGF), b27 and N2 supplements in a low adherence system to form tumour spheres that exhibit self-renewal and tumourigenicity [53]. The culture medium conditions used for this assay provide an appropriate niche environment required for forming spheres and maintaining the stemness of CSCs [53]. For instance, EGF maintains pluripotency in neuroglioma stem cells, whereas bFGF is critical for the formation of tumour spheres [54]. Similarly, b27 is vital in sphere formation and maintaining characteristics of CSCs, and N2 supplement supports the proliferation of tumour spheres [54,55]. The CSCs enriched by this method are capable of forming three-dimensional spheroids with a heterogeneous population of progenitor cells that can differentiate into multiple cell phenotypes [56]. Notably, the sphere-forming culture method is based on the ability of CSCs to grow as anchorage-independent three-dimensional spheres and is not dependent on CSC marker expression [57]. The sphere-forming assay has been increasingly utilised in various tumours, including HCC for isolating, enriching, maintaining or expanding the potential CSC subpopulations [53,55,57,58,59].

In this study, the sphere-forming assay was used to examine the stemness and chemoresistance properties with several in vitro assays. Furthermore, we evaluated the relationship between EMT and immune checkpoint molecule expression in the CSC population. We also demonstrated that human HCC-derived CSCs exhibit mesenchymal traits with increased expression of immune checkpoints. Thus, we utilized the combined targeting of EMT and immune checkpoints to enhance the cytotoxicity of Sorafenib against resistant HCC-derived CSCs.

## 2. Materials and Methods

### 2.1. Cell Culture and Reagents

The human HCC cell lines PLC/PRF/5 (85061113) and HepG2 (85011430) were purchased from CellBank Australia. The human HCC cell line Hep3B was kindly provided by Prof. V. Nathan Subramaniam, The Queensland University of Technology, Australia. All cell lines were mycoplasma-tested using the MycoAlert test (Abm, Richmond, BC, Canada) and cultured in Dulbecco’s modified Eagle’s medium (DMEM) (Thermofisher, Victoria, Australia) with 10% fetal bovine serum (FBS) (Gibco, Victoria, Australia) and 1% penicillin/streptomycin (P/S) (Thermofisher, Victoria, Australia) and incubated in a humidified atmosphere with 5% CO_2_ at 37 °C [57]. Sorafenib was procured from Selleckchem.com, Australia. The TGF-*β* receptor kinase inhibitor, SB431542 (Sigma, New South Wales, Australia), was used at a concentration of 2 µM.

### 2.2. Three-Dimensional Sphere Enrichment Assay

A serum-free stem-cell-conditioned medium was prepared with 1:1 mixture of DMEM/HAM’s F-12 (Thermofisher, Victoria, Australia) supplemented with 1% pencillin/streptomycin (P/S) (Thermofisher, Victoria, Australia), 10 ng/mL human recombinant basic fibroblast growth factor (hrbFGF) (Peprotech, Lonza, Victoria, Australia), 20 ng/mL human recombinant epidermal growth factor (hrEGF) (Peprotech, Lonza, Victoria, Australia), 2% B27 supplement without vitamin A (Thermofisher, Victoria, Australia) and 1% N2 supplement (Thermofisher, Victoria, Australia) [58]. The parental adherent cells were dissociated with Trypsin-EDTA (Thermofisher, Australia) and the detached cells were washed three times with 1XPBS to remove serum. Approximately 5000 cells/well were seeded in a 6-well ultra-low attachment plate and cultured with serum-free stem-cell-conditioned medium at 37 °C under a humidified atmosphere with 5% CO_2_ in air [57]. The spheres were photographed under an inverted microscope equipped with a digital camera (Olympus DP21, Tokyo, Japan) and collected after 5 days [57].

### 2.3. Colony-Forming Assay

Both adherent cells and spheres were enzymatically dissociated with Trypsin-EDTA and stained with Trypan Blue dye (Thermofisher, Australia) to determine cell viability and cell count. Approximately 100 cells were seeded in 6-well plates with DMEM medium. After 7 days, the colony-forming ability was assessed after staining with Crystal Violet (Sigma, Australia). Representative views were photographed under an inverted microscope equipped with a digital camera (Olympus DP21, Japan).

### 2.4. RNA Extract and cDNA Synthesis

RNA was isolated as previously described using an Isolate II Bioline RNA synthesis kit (Bioline, New South Wales, Australia) as per the manufacturer’s protocol [57]. Nanodrop 2000c (Thermofisher, Australia) was used to confirm the quantity and purity of RNA. cDNA was synthesised by reverse transcribing 1 µg RNA using a Bioline SensiFAST cDNA synthesis kit (Bioline, Australia).

### 2.5. Quantitative Reverse Transcription-PCR (qRT-PCR)

qRT-PCR was performed using Lo-ROX SYBR Green (Bioline, Australia) on a ViiA7 Applied Biosystems Real-Time PCR system as previously described [57]. Beta-Actin (*ActB*) was used as an internal control. The primer sequences were previously reported [60] and listed in Table 1. Expression levels are presented as copies of the target gene per 10,000 copies of *ActB*. The 2ΔΔCt method was used for data analysis, where *ActB* was assigned as the housekeeping gene. In this 2ΔΔCt method, target gene expression was normalised to *ActB* expression and data are presented as copies of the target gene per 10,000 copies of *ActB*.

### 2.6. Western Blot Analysis

This method was performed as previously described [60]. Briefly, cells cultured and treated in 6-well plates. Cells were lysed using RIPA buffer (Thermofisher, Victoria, Australia) with Complete (Roche, New South Wales, Australia) and phosSTOP (Roche, New South Wales, Australia) protease and phosphatase inhibitors at 4 °C to extract the total protein. The total protein was quantified with a Pierce BCA protein assay kit (Thermofisher, Victoria, Australia). A total of 10 µg of protein was used for separation by electrophoresis (SDS-PAGE) in a polyacrylamide gel in the presence of sodium dodecyl sulphate (SDS) and transferred to a polyvinylidene difluoride film (PVDF) membrane. Following blocking with 5% skim milk in Tris-buffered saline containing 0.1% Tween 20 (TBS-T), the membranes were exposed to primary antibodies at 4 °C overnight. Next, the membranes were incubated with HRP-conjugated secondary antibodies and SuperSignal West Femto Maximum Sensitivity Substrate (Thermofisher, Victoria, Australia) was used to detect the protein adhered to the membrane. Glyceraldehyde 3-phosphate dehydrogenase (GAPDH) and Beta-Actin were used as the housekeeping controls. Image Quant LAS 500 was used to capture images and Image Studio Lite Ver 5.2 software (VWR, International, Radnor, PA, USA) was used for quantification. The antibodies used were previously reported [60] and are listed in Table 2.

### 2.7. Transwell Migration Assay

Migration assay was performed with transwell chambers (8 µm pore size, Corning, Australia). Cells grown as spheres and monolayer were enzymatically dissociated to single cells with Trypsin-EDTA treatment. Trypan Blue dye (Thermofisher, Australia) staining was performed to determine cell viability and cell count. 1 × 10^5^ of harvested cells from sphere or monolayer culture were plated into the upper chamber in a serum-free DMEM culture medium. A total of 500 µL of DMEM culture medium with 10% FBS was added into the lower chamber as the chemoattractant. Following 24 h of incubation at 37 °C, cells were treated with 4% paraformaldehyde (Fisher Scientific, Victoria, Australia) for 15 min for fixation. Next, cells were stained with 0.1% Crystal Violet (Sigma-Aldrich, New South Wales, Australia). The cells were removed from the upper chamber. Cells migrated onto the lower chamber were photographed under an inverted Olympus DP21 microscope equipped with a digital camera. For the quantification of cell migration, the Crystal Violet staining on the transwell membrane was extracted using 5% SDS and a 570 nm wavelength was used to measure absorbance [61].

### 2.8. Proliferation Assay

1 × 10^3^ cells were seeded in 96-well plates and treated as indicated. Cell proliferation was quantified with the CellTitre 96 Aqueous one solution cell proliferation assay (MTS) from Promega, Australia according to the manufacturer’s protocol.

### 2.9. siRNA Transfection

Cells were transfected at 50% confluency for transient siRNA transfection with Lipofectamine RNAiMAX (Invitrogen, Victoria, Australia) and 10 nM final concentration of a control siRNA (4390843) (Thermofisher, Australia) and two distinct silencer select siRNAs targeting PD-L1 (s26547 and s26548) (Thermofisher, Victoria, Australia) and CD73 (s9735 and s9734) (Thermofisher, Victoria, Australia) according to manufacturer’s protocol. Cells were incubated with siRNA complex for 72 h and then collected for further experiments.

### 2.10. Flow Cytometry

Cells were plated and treated as indicated. Cells grown as spheres and monolayer were enzymatically dissociated to single cells with Trypsin-EDTA. Harvested cells were then washed in cold 1XPBS. The apoptosis induced by different treatment approaches was demonstrated using FITC Annexin V/Dead Cell Apoptosis Kit (Invitrogen, Victoria, Australia). The Annexin V is used for detecting cellular apoptosis, and propidium iodide (PI) is used for detecting necrotic or late apoptotic cells. For this assay, the 1× PBS washed cells were resuspended in 100 µL of 1× annexin-binding buffer per assay at density of approximately 1 × 10^6^ followed by addition of 5 µL of FITC annexin V and 1 µL of 100 μg/mL PI. After the incubation period of 15 min at room temperature, 400 µL of 1× annexin-binding buffer was gently mixed and the samples were kept on ice. The cells were analysed using flow cytometry (Fortessa X-20, BD Biosciences, San Jose, CA, USA). BD FACSDiva software was used for data acquisition and FlowJo software was used for data analysis. The data obtained were plotted in two-dimensional dot plots where PI on the y-axis was plotted against Annexin V-FITC on the x-axis. These plots were divided in four quadrants. The quadrant Q1 represents apoptotic cells that were PI positive and Annexin V negative, the quadrant Q2 represents late apoptotic cells that were PI and Annexin V positive, the quadrant Q3 represents apoptotic cells that were PI negative and Annexin V positive and the quadrant Q4 represents viable cells that were negative for both Annexin V and PI.

### 2.11. Statistical Analysis

Prism software version 8.00 (GraphPad Software Inc., San Diego, CA, USA) was used for statistical analyses. Gene expression differences between control and Sorafenib-treated cells were analysed using Student’s *t*-test. For multiple comparisons, one-way analysis of variance (ANOVA) followed by Sidak’s multiple comparisons test was used. Two-way ANOVA followed by Tukey’s multiple comparisons test was performed for statistical analysis for cell viability assay. All experiments were repeated at least three times and representative results are presented. The results are presented as mean ± standard error of mean. Statistical significance was set at *p* < 0.05. Error bars indicate standard error of the mean (SEM).

## 3. Results

### 3.1. Human HCC-Derived Spheres Exhibit Stem-Cell-Like Features

**Figure 1:**
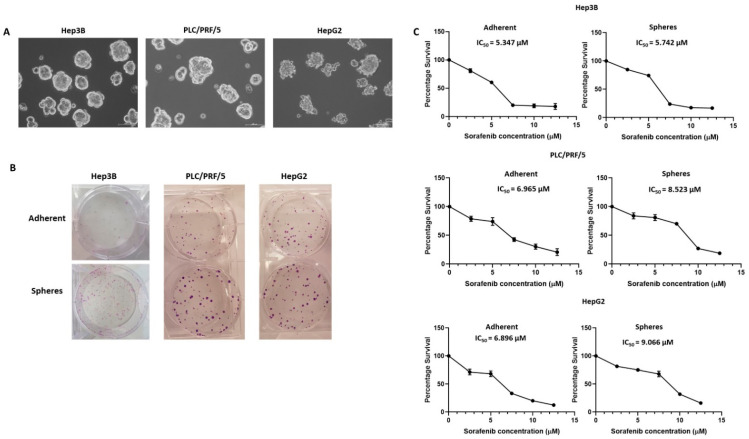
Enrichment of human HCC-derived spheres. (**A**) Human HCC cell lines Hep3B, PLC/PRF/5 and HepG2-derived anchorage independent spheres in stem-cell-conditioned culture medium (scale bar = 100 µm). (**B**) Colony-forming assay revealed a higher number of colonies formed from HCC-derived spheres compared to parental cells. (**C**) Cell viability assay demonstrated the increased Sorafenib resistance of HCC-derived spheres with higher IC_50_ values compared to parental cells after 72 h of increasing doses of Sorafenib treatment.

Human HCC cell lines, Hep3B, PLC/PRF/5 and HepG2 were used for the sphere-forming assay to enrich CSCs from adherent parental cells. All three cell lines formed anchorage-independent three-dimensional spheres free of any adherent cells when cultured in serum-free stem-cell-conditioned medium by Day 5 (Figure 1A). HCC parental cells termed adherent were cultured as conventional 2D monolayers without growth factor supplements. As previously reported, adherent cells were used for control and compared with spheres in the following experiments [57,62,63,64].

To examine the self-renewal and proliferative abilities of human HCC-derived spheres, we utilised a colony-forming assay to compare the colony-forming ability between adherent cells and spheres. We demonstrated that spheres proliferated rapidly with a higher number of colonies in comparison to parental adherent cells for all three cell lines (Figure 1B), indicating that three-dimensional spheres are more proliferative and could be vital in the maintenance of tumour growth.

Furthermore, proliferation assay was used to determine the chemoresistant property of anchorage-independent spheres. The sensitivity of three-dimensional spheres versus parental adherent cells to Sorafenib were examined and IC_50_ values calculated. Notably, the spheres showed higher resistance to Sorafenib compared to adherent cells with higher IC_50_ values for all cell lines examined (Figure 1C). The IC_50_ values for Hep3B adherent cells and spheres were 5.347 and 5.742 µM, respectively, whereas IC_50_ values for PLC/PRF/5 adherent and spheres were 6.965 and 8.523 µM, respectively. Similarly, the IC_50_ values for HepG2 adherent cells and spheres were 6.896 and 9.066 µM, respectively. Based on these findings, we refer to HCC-derived CSCs as Sorafenib-resistant CSCs.

**Figure 2:**
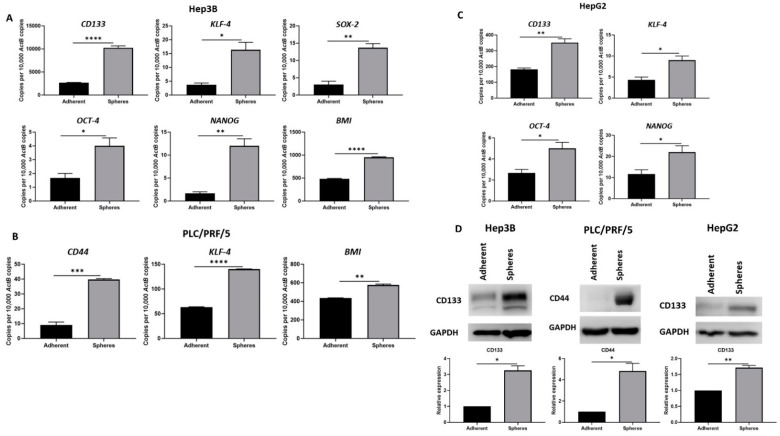
Human HCC-derived spheres exhibit stem-cell-like features. (**A**) qRT-PCR demonstrated upregulation of stem cell markers CD133, KLF-4, SOX-2, OCT-4, NANOG, and BMI in Hep3B-derived spheres compared to adherent Hep3B cells. (**B**) PLC/PRF/5-derived spheres exhibited increased expression of CD44, KLF-4 and BMI compared to adherent cells as shown by qRT-PCR. (**C**) qRT-PCR revealed higher expression of CD133, KLF-4, OCT-4 and NANOG in HepG2-derived spheres compared to parental cells. (**D**) Increased CD133 expression in Hep3B and HepG2-derived spheres and upregulation of CD44 in PLC/PRF/5-derived spheres compared to adherent cells. GAPDH was the loading control. Detailed information about Western Blot can be found at Supplementary Materials. (*n* = 3, * *p* < 0.05, ** *p* < 0.01, *** *p* < 0.005, **** *p* < 0.001).

To further characterise the enriched spheres, we assessed the putative stem-cell-associated marker expression. An equal number of cells was plated for adherent and sphere cultures. The cells were collected at Day 5 for RNA and protein extraction. As demonstrated by qRT-PCR, stem-cell-associated genes *CD133*, *KLF-4*, *SOX-2*, *OCT-4*, *NANOG* and *BMI* were elevated in Hep3B-derived spheres compared to adherent cells (Figure 2A). Similarly, we observed higher expression of *CD44*, *KLF-4* and *BMI* in PLC/PRF/5-derived spheres compared to adherent cells (Figure 2B) and increased expression of *CD133*, *KLF-4*, *OCT-4* and *NANOG* in HepG2-derived spheres compared to adherent cells (Figure 2C). The upregulation of putative CSC marker CD133 in spheres compared to adherent cells was further confirmed by Western blot analysis in Hep3B and HepG2 cells. Similarly, upregulation of CD44 was observed in spheres derived from PLC/PRF/5 compared to parental adherent cells (Figure 2D). These results support the enrichment of the CSC population with sphere-forming assay.

### 3.2. Anchorage-Independent Spheres Exhibit Mesenchymal Phenotype

**Figure 3:**
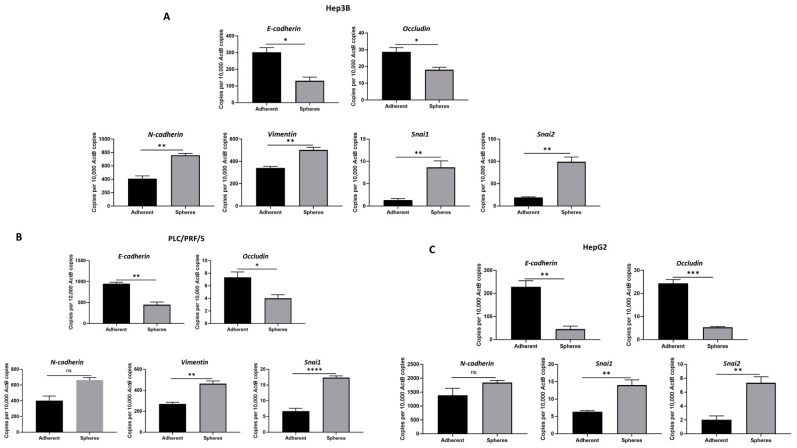
Human HCC-derived spheres exhibit mesenchymal phenotype. qRT-PCR demonstrated (**A**) a decrease in epithelial markers E-cadherin and Occludin and an increase in mesenchymal markers N-cadherin, Vimentin, Snai1 and Snai2 expression in Hep3B-derived spheres compared to adherent cells, (**B**) a reduction in E-cadherin and Occludin expression and an elevation in N-cadherin, Vimentin and Snai1 expression in PLC/PRF/5-derived spheres compared to adherent cells, and (**C**) downregulation of E-cadherin and Occludin and upregulation of N-cadherin, Snai1 and Snai2 in HepG2-derived spheres compared to adherent cells. (*n* = 3, * *p* < 0.05, ** *p* < 0.01, *** *p* < 0.005, **** *p* < 0.001, ns: not significant).

To explore the relationship between EMT and CSCs, we examined the EMT features related to HCC-derived CSCs. The epithelial markers *E-cadherin* and *Occludin* showed decreased expression, whereas the mesenchymal markers *N-cadherin*, *Vimentin*, *Snai1* and *Snai2* showed increased expression in Hep3B-derived CSCs compared to adherent cells (Figure 3A), as demonstrated by qRT-PCR. Similarly, we observed a decrease in E-*cadherin* and *Occludin* expression along with an increase in *N-cadherin*, *Vimentin* and *Snai1* expression in PLC/PRF/5-derived CSCs compared to adherent cells (Figure 3B). In addition, HepG2-derived CSCs also demonstrated reduced expression of epithelial markers *E-cadherin* and *Occludin* with an elevated expression of mesenchymal markers *N-cadherin*, *Snai1* and *Snai2* (Figure 3C).

**Figure 4:**
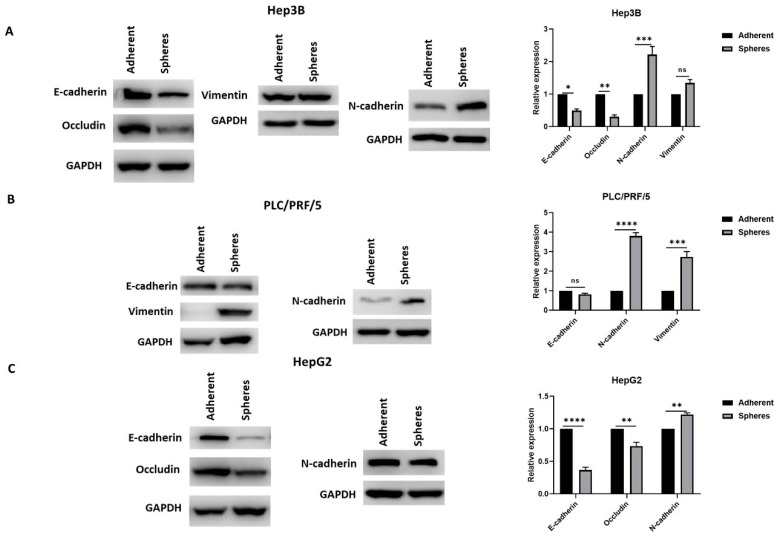
Human HCC-derived spheres exhibit mesenchymal phenotype. Western blot analysis revealed (**A**) a decrease in E-cadherin and Occludin expression and an increase in N-cadherin and Vimentin expression in Hep3B-derived spheres compared to adherent cells, (**B**) reduced expression of E-cadherin and elevated expression of N-cadherin and Vimentin in PLC/PRF/5-derived spheres compared to adherent cells, and (**C**) reduced expression of E-cadherin and Occludin and elevated expression of N-cadherin in HepG2-derived spheres compared to adherent cells. Detailed information about Western Blot can be found at Supplementary Materials. (*n* = 3, * *p* < 0.05, ** *p* < 0.01, *** *p* < 0.005, **** *p* < 0.001, ns: not significant).

This association of stem cell phenotype with EMT phenotype was further confirmed by Western blot analysis wherein epithelial markers were downregulated and mesenchymal markers were upregulated in CSCs compared to adherent cells in Hep3B (Figure 4A), PLC/PRF/5 (Figure 4B) and HepG2 (Figure 4C).

**Figure 5:**
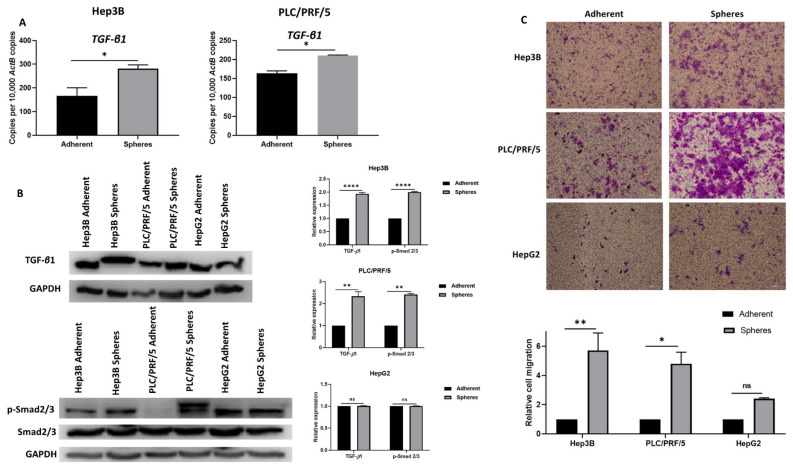
Human HCC-derived spheres exhibit mesenchymal phenotype. (**A**) qRT-PCR demonstrated an upregulation of TGF-*β*1 in Hep3B and PLC/PRF/5-derived spheres compared to adherent cells. (**B**) Western blot analysis revealed an upregulation of TGF-*β*1 and p-Smad2/3 in Hep3B and PLC/PRF/5-derived spheres compared to adherent cells and no upregulation of TGF-*β*1 and p-Smad2/3 in HepG2-derived spheres compared to adherent cells. GAPDH was the loading control. Detailed information about Western Blot can be found at Supplementary Materials. (**C**) Transwell migration assay demonstrated higher motility of human HCC-derived spheres compared to adherent cells (scale bar = 500 µm). The number of motile cells on the transwell membrane was quantified by measuring the absorbance of Crystal Violet staining. (*n* = 3, * *p* < 0.05, ** *p* < 0.01, **** *p* < 0.001, ns: not significant).

To further confirm EMT activation in CSCs, we assessed the activation of transforming growth factor (TGF)-*β*1, a potent inducer of EMT. We observed elevated expression of TGF-*β*1 in Hep3B and PLC/PRF/5-derived CSCs compared to adherent cells, as demonstrated by qRT-PCR. However, no upregulation of TGF-*β*1 was observed in HepG2 spheres (Figure 5A). The activation of TGF-*β*1 signalling in CSCs was further confirmed by the phosphorylation of Smad2/3 using Western blot analysis (Figure 5B). These findings suggest the activation of Smad-dependent TGF-*β*1 signalling in HCC-derived CSCs.

We utilised transwell migration assay to examine the migratory ability of HCC-derived CSCs compared to adherent cells. We observed that HCC-derived CSCs exhibited higher migratory ability (Figure 5C). Together, these findings suggest that anchorage-independent spheres exhibit the mesenchymal phenotype and may be modulated by EMT, particularly by the activation of Smad-dependent TGF-*β*1 signalling.

### 3.3. Inhibition of EMT with SB431542 Can Enhance Cytotoxicity of SORAFENIB

**Figure 6:**
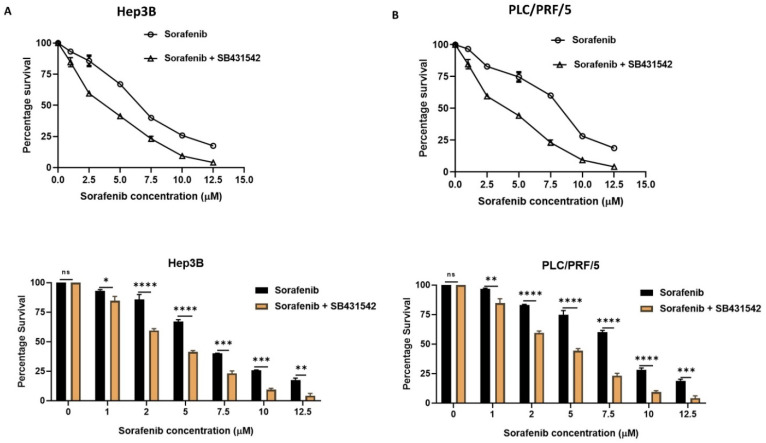
EMT inhibition with SB431542 enhanced the cytotoxic effects of Sorafenib. Cell viability assay revealed increased cytotoxicity of Sorafenib when combined with SB431542 in comparison to Sorafenib alone in (**A**) Hep3B-derived spheres and (**B**) PLC/PRF/5-derived spheres. (*n* = 3, * *p* < 0.05, ** *p* < 0.01, *** *p* < 0.005, **** *p* < 0.001, ns: not significant).

Given our observation that HCC-derived CSCs exhibit a mesenchymal phenotype and have a close association with EMT during the activation of TGF-*β*1 signalling in Hep3B and PLC/PRF/5-derived spheres, we hypothesise that EMT may be responsible for maintaining tumour growth and therapy resistance in Hep3B and PLC/PRF/5-derived CSCs. Thus, we utilised SB431542, a specific inhibitor of TGF-*β* receptor kinase, to treat Hep3B and PLC/PRF/5-derived CSCs with a concentration of 2 µM for a period of 72 h to assess the variation in cytotoxic effects of Sorafenib alone versus in combination with SB431542 (inhibition of TGF-*β*1-induced EMT). The proliferation assay demonstrated that the cytotoxic effects of Sorafenib in HCC-derived CSCs was enhanced when combined with SB431542 in comparison to Sorafenib alone (Figure 6A,B), suggesting better therapeutic efficacy of Sorafenib when combined with an EMT inhibitor.

### 3.4. CSCs Overexpress Immune Checkpoint Molecules

**Figure 7:**
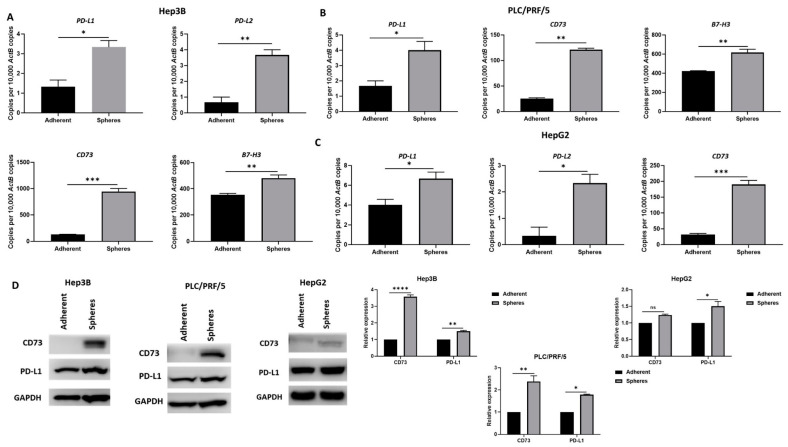
Human HCC-derived spheres overexpress immune checkpoint molecules. (**A**) qRT-PCR demonstrated upregulation of PD-L1, PD-L2, CD73 and B7-H3 in Hep3B-derived spheres compared to adherent cells. (**B**) PLC/PRF/5-derived spheres overexpress PD-L1, CD73 and B7-H3 compared to adherent cells, and (**C**) HepG2-derived spheres exhibited increased expression of PD-L1, PD-L2 and CD73 compared to adherent cells, as revealed by qRT-PCR. (**D**) Western blot analysis demonstrated an increase in CD73 and PD-L1 expression in Hep3B, PLC/PRF/5 and HepG2-derived spheres compared to adherent cells. GAPDH was the loading control. Detailed information about Western Blot can be found at Supplementary Materials. (*n* = 3, * *p* < 0.05, ** *p* < 0.01, *** *p* < 0.005, **** *p* < 0.001, ns: not significant).

To gain a better understanding of the role of the immune checkpoint in the Sorafenib resistance mechanism in HCC, we evaluated the expression of immune checkpoint molecules in HCC-derived CSCs by qRT-PCR and Western blot analysis. In particular, we sought to analyse the expression of ICIs in CSCs that were associated with poor HCC patient prognosis, as reported in our previous studies [60,65]. Higher expression of *PD-L1*, *PD-L2*, *CD73* and *B7-H3* was observed in Hep3B-derived CSCs compared to adherent cells (Figure 7A). Similarly, PLC/PRF/5-derived CSCs exhibited an increase in *PD-L1*, *CD73* and *B7-H3* expression compared to adherent cells (Figure 7B), whereas HepG2-derived CSCs exhibited increased expression of *PD-L1*, *PD-L2* and *CD73* compared to adherent cells (Figure 7C). The increase in the expression of immune checkpoint molecules, PD-L1 and CD73 in CSCs was further confirmed by Western blot analysis in all cell lines examined (Figure 7D).

### 3.5. Combination of Immune Checkpoint and EMT Inhibition Can Sensitise Human HCC-Derived CSCs to Sorafenib Treatment

As the inhibition of EMT enhances the cytotoxic effects of Sorafenib, resulting in the re-sensitising of CSCs to Sorafenib, we further evaluated the combined effects of EMT inhibition along with immune checkpoint inhibition on the therapy resistance of CSCs to Sorafenib. The expression of immune checkpoint molecules PD-L1 and CD73 were depleted with RNA interference. We demonstrated knockdown of PD-L1 using two different siRNAs in our previous study [61]. In this study, we also observed the effective knockdown of CD73 with two siRNA targeting CD73 in both Hep3B and PLC/PRF/5 cells as confirmed by qRT-PCR and Western blot analysis (Figure S1A,B).

**Figure 8:**
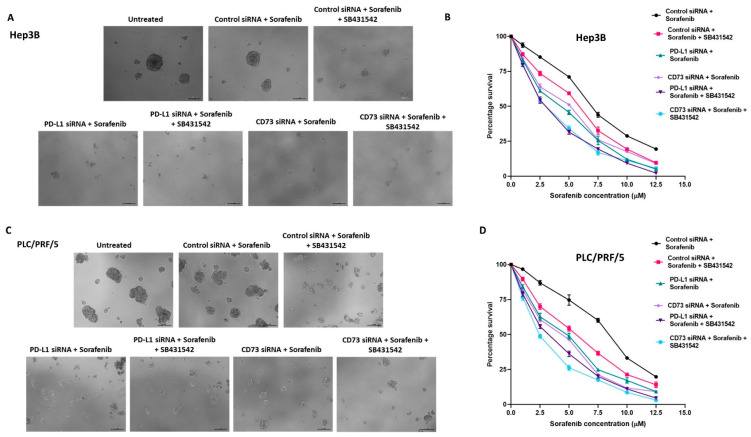
Combined targeting of EMT and immune checkpoint molecules enhanced the cytotoxicity of Sorafenib against human HCC-derived spheres. (**A**) Day 5 images of Hep3B-derived spheres revealed a decreased sphere size as a result of increased cytotoxicity of Sorafenib when combined with SB431542 and PD-L1 or CD73 siRNA compared to Sorafenib alone (scale bar = 200 µm). (**B**) Cell viability assay revealed reduced viability of Hep3B-derived spheres following combination treatment of Sorafenib with SB431542 and PD-L1 siRNA or CD73 siRNA compared to Sorafenib alone. (**C**) Day 5 images of PLC/PRF/5-derived spheres demonstrated increased cytotoxicity of Sorafenib when combined with SB431542 and PD-L1 siRNA or CD73 siRNA compared to Sorafenib alone with decreased sphere size (scale bar = 200 µm). (**D**) Cell viability assay revealed reduced viability of PLC/PRF/5-derived spheres following combination treatment of Sorafenib with SB431542 and PD-L1 siRNA or CD73 siRNA compared to Sorafenib alone.

Further, we assessed the effects of EMT inhibitor, SB431542, alone and in combination with either PD-L1 or CD73 knockdown on the cytotoxicity of Sorafenib. The proliferation assay demonstrated that the number and size of spheres formed at Day 5 were significantly reduced when Sorafenib was combined with SB431542 and/or PD-L1 or CD73 siRNA in Hep3B (Figure 8A,B). Similarly, we noted that the reduction in the number and size of spheres when combined with Sorafenib was combined with SB431542 and/or PD-L1 siRNA or CD73 siRNA in PLC/PRF/5 (Figure 8C,D). We also observed better therapeutic effects of CD73 knockdown on Sorafenib and SB431542 in comparison to PD-L1 knockdown.

Next, we examined whether the treatment of HCC spheres with Sorafenib or SB431542 affected the expression of stemness markers and immune checkpoint molecules. We observed downregulation of stemness markers *CD133*, *KLF-4*, *SOX2*, *OCT-4*, *NANOG* and *BMI* and immune checkpoint molecules *CD73* and *PD-L1* in Hep3B-derived spheres treated with Sorafenib compared with untreated Hep3B-derived spheres (Figure S2A,B). PLC/PRF/5-derived spheres showed downregulation of *CD44* and *BMI* along with upregulation of *CD73* and *PD-L1* upon Sorafenib treatment (Figure S3A,B). SB431542-treated Hep3B spheres showed downregulation of stemness markers *CD133, KLF-4, SOX2, OCT-4, NANOG* and *BMI* and immune checkpoint molecules *CD73* and *PD-L1* (Figure S4A,B). SB431542-treated PLC/PRF/5-derived spheres downregulated the expression of stemness markers *CD44* and *BMI* with upregulated expression of *KLF-4* along with upregulation of *CD73* and *PD-L1* compared with untreated spheres (Figure S5A,B). Knockdown of either CD73 or PD-L1 in Hep3B spheres decreased the expression of stemness markers *CD133*, *KLF4*, *SOX2*, *OCT-4*, *NANOG* and *BMI* compared with control siRNA-treated spheres (Figure S6). Similarly, treatment of PLCPRF5-derived spheres with either CD73 or PD-L1 siRNA resulted in the downregulation of stemness markers *CD44* and *BMI* (Figure S7).

**Figure 9:**
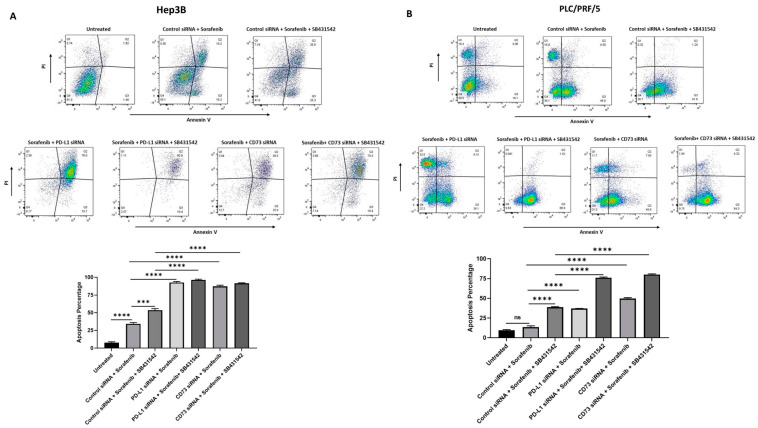
Combined targeting of EMT and immune checkpoint molecules enhanced Sorafenib-induced apoptosis in human HCC-derived spheres. Flow cytometric analysis of Annexin V/Propidium Iodide staining revealed increased percentage of apoptotic cells following combination treatment of Sorafenib with SB431542 and PD-L1 or CD73 siRNA compared to Sorafenib alone in (**A**) Hep3B-derived spheres and (**B**) PLC/PRF/5-derived spheres. (*n* = 3, *** *p* < 0.005, **** *p* < 0.001, ns: not significant).

To further validate the increased cytotoxic effects of Sorafenib on HCC-derived CSCs with combined targeting of EMT and immune checkpoint molecules, we used flow cytometry analysis to examine apoptosis induced by Sorafenib alone versus combination with EMT inhibitor and/or immune checkpoint inhibitors. We observed that the percentage of apoptotic spheres increased with a combination therapy of SB431542 and immune checkpoint inhibitors (PD-L1 siRNA or CD73 siRNA) with Sorafenib compared to Sorafenib alone in both Hep3B (Figure 9A) and PLC/PRF/5-derived (Figure 9B) CSCs. These findings suggest that the combined targeting of EMT and immune checkpoint molecules can enhance the cytotoxic effects of Sorafenib or overcome the resistance of HCC-derived CSCs to Sorafenib, resulting in effective chemotherapeutic outcome in HCC.

## 4. Discussion

The therapeutic benefit of Sorafenib, the standard clinical treatment for patients with unresectable advanced HCC, is limited primarily due to the acquisition of resistance to Sorafenib [6,7,8]. Thus, there is a pressing need to identify important mechanisms responsible for Sorafenib resistance in HCC and to develop better treatment alternatives to improve the efficacy of Sorafenib and overcome Sorafenib resistance. Herein, we report that CSCs, the chemoresistant subpopulation of HCC tumour are closely associated with EMT and overexpress immune checkpoint molecules. This study suggests a critical role of EMT, immune checkpoint molecules and CSCs together in orchestrating Sorafenib resistance. We thus utilised combined therapeutic approach to inhibit TGF-*β*1-induced EMT with SB431542 and immune checkpoint inhibition with PD-L1 and CD73 knockdown to improve the therapeutic effectiveness of Sorafenib and to overcome Sorafenib resistance in HCC-derived CSC subpopulation.

CSCs contribute to the development, recurrence, metastasis and maintenance of HCC [38,41,66]. Studies have identified several factors that contribute to Sorafenib resistance in HCC, and CSCs are one of the clinical factors closely associated with drug resistance [11,15,16]. Studies have revealed that the HCC tumour sub-populations that are highly resistant to cancer therapies possess more CSCs [66,67]. Thus, targeting CSCs is key to improving therapeutic outcome in cancer treatment. Initially, CSCs were isolated and identified based on the side population method by flow cytometry using DNA-intercalating dye Hoechst 33342 or the magnetic activated cell sorting (MACS) and fluorescence activated cell sorting (FACS) based on cell surface markers [68,69,70]. In the present study, we have utilised a sphere-forming assay for enriching the cancer stem cell population in serum-free culture medium that provides the appropriate niche environment required for the maintenance of the stemness of CSCs without the pre-selection of specific cell surface markers [53]. Previous studies have demonstrated that the sphere-forming assay can enrich CSCs by evaluating a combination of CSC marker expression and assessing the self-renewing and proliferative abilities of CSCs using limited dilution and expansion assays [53]. The present study revealed that spheres enriched for CSCs possess stem-cell-like features with higher proliferation and chemoresistant abilities consistent with findings from other studies [53,55,58].

EMT is another factor contributing to therapy resistance in several cancers, including HCC [28,32,34,71]. Studies have also revealed that EMT plays an important role in the generation and maintenance of CSC population in cancers, including HCC [32,35,36,37]. Moreover, EMT and CSCs are reported to be mechanistically linked and contribute to the development of Sorafenib resistance [12,32]. Our study demonstrated that human HCC-derived CSCs exhibit a mesenchymal phenotype. Similarly, many studies have reported that EMT activation is closely associated with the gain of stem-cell-like features [35,72,73,74]. A study by Jing et al. utilised the Oncomine database to demonstrate the correlation between EMT and CSC markers in HCC [72]. The study also revealed that HIF-1α-driven EMT mediated the acquisition of stem-cell-like features such as expression of CSC markers, stronger colony-forming ability and sphere formation ability in human HCC cells [72]. We have previously shown a similar association between the expression of EMT markers and CSCs in murine HCC and normal hepatocyte cell lines [57].

CSCs are known to possess immune-evasive properties through a reduction in major histocompatibility complex class I (MHC I) and molecules for antigen presentation in cancers such as colon cancer [75], melanoma [76] and breast cancer [77]. The association between immune suppression and CSC is well studied in glioblastoma, where studies have demonstrated an important role of CSCs in the initiation of immune suppression with interplay between CSCs and immune cells changing from stimulatory to inhibitory status throughout tumourigenesis [78,79,80]. Another study by Ozawa et al. revealed that glioma stem cells exhibit higher indoleamine 2,3-dioxygenase 1 (IDO-1) expression, resulting in immunosuppression and therapy resistance [81]. Furthermore, studies have demonstrated that enrichment of PD-L1 expression in CSCs facilitates immune evasion in lung [82,83] and head and neck cancer [83]. This association between CSCs and immune evasion is not extensively studied in HCC. Nishida et al. revealed that the expression of stem cell markers CK19 and SALL4 was positively correlated with the expression of PD-L1 in HCC patients [84]. In addition to the well-documented role of PD-L1 in suppressing the anti-tumour immune response by interacting with PD1 receptor on T-cells and subsequently inactivating T-cells, recent studies have reported a distinct tumour-intrinsic role for PD-L1 in regulating CSCs, EMT, motility, metastasis and drug resistance, including resistance to Sorafenib [61,85]. Studies have been conducted on tumour cells in the absence of T-cells to evaluate the intrinsic functions of immune checkpoints. Immune-independent studies of PD-L1 in breast cancer, melanoma and ovarian cancer facilitate the generation of CSCs [86,87]. Similarly, immune-independent studies of CD73 have reported that it promoted HCC progression and metastasis by activating the transcription of SOX9 and also increases its protein stability in an AKT/glycogen synthase kinase (GSK)3β-dependent manner, which sustained the stemness of HCC cells [88,89].

Our study is the first to report a higher expression of immune checkpoints in enriched HCC-derived CSCs compared to adherent cells. Given the increased expression of several immune checkpoint molecules such as PD-L1, CD73, B7-H3 and others in HCC-derived CSCs, it is conceivable that CSCs exhibit immune-evasive features that may contribute to Sorafenib resistance. Further studies using RNA sequencing technology to profile HCC-derived CSCs and to identify novel immune regulatory molecules expressed on these CSCs will be more informative.

Our study demonstrated a significant association between CSCs and EMT along with immune checkpoint molecules. These findings suggest that HCC-derived CSCs exhibit Sorafenib resistance by immune evasion through enhanced expression of immune checkpoints such as PD-L1 and CD73 following the induction of EMT. Whilst we observed elevated expression and activation of TGF-*β*1 in Hep3B and PLC/PRF/5-derived CSCs, no upregulation of TGF-*β*1 was observed in HepG2 spheres. Although all three cell lines had similar E-cad levels and expression of stemness markers, the differences in TGF-*β* expression among Hep3b, PLC/PRF/5 and HepG2 spheres may be due to the intrinsic property of the cell lines. Frequent aberrations in TGF-*β* superfamily at both the transcriptomic level and the genomic level have been reported to occur in HCC leading to either activation or inactivation of TGF-*β* signalling [90]. This study implicates several genetic mutations or expression levels of different genes, such as *SIRT1-6* and *SPTBN1*, that may contribute to alterations in TGF-*β* signalling [90]. Further studies are needed to examine the specific reason for differences in TGF-*β* signalling across the three HCC-derived spheres in this study.

We have previously reported that cytokine-driven EMT in HCC cells can regulate the expression of immune checkpoints, including PD-L1 and CD73 [60]. The present study further demonstrates acquisition of Sorafenib resistance following activation of EMT which results in the regulation of immune checkpoint molecules and the maintenance of the CSC population. Thus, we hypothesised that targeting EMT and immune checkpoint molecules can be a better therapeutic alternative to effectively target the therapy-resistant CSC population, resulting in the effective elimination of HCC tumour bulk. Consequently, we utilised SB431542 to inhibit TGF-*β*1-induced EMT and siRNA against PD-L1 and CD73 for immune checkpoint inhibition as combination therapy to overcome resistance to Sorafenib in the CSC population. The present study reported the effective targeting of CSCs with reduced proliferation and increased apoptosis following combination treatment via the enhanced cytotoxicity of Sorafenib. We noted that SB431542 treatment induced a pronounced early-stage apoptosis in PLC/PRF/5 cells when compared with Hep3B cells. We speculate that these differences may be attributed to the intrinsic difference of the cell line itself. This heterogeneity noted in cell lines often mirrors heterogeneity observed across HCC patients. Further investigation into whether the EMT inhibitor-, ICIs- and Sorafenib-induced apoptosis is mediated via either the intrinsic pathway by the mitochondrial cascade involving Bcl-2/Bax family proteins, active caspases, and Akt signalling or the extrinsic pathway by the expression of surface death receptors will be pivotal to understand the mechanisms of actions employed by these agents [91].

Our study is the first to demonstrate the combined targeting of EMT and immune checkpoint molecules as an effective therapeutic treatment to overcome Sorafenib resistance. Furthermore, either monoclonal antibodies or siRNAs directed against immune checkpoints can be used for targeting CSCs. Whilst monoclonal antibodies can block immune checkpoint interaction, we sought to examine if the loss of expression of immune checkpoints with siRNA can reveal insights into molecular mechanisms such as EMT and TGF-*β* signal transduction. Further studies with monoclonal antibodies targeting CD73 and PD-L1 can be tested to corroborate the findings from this study with siRNAs.

Monotherapy treatment of Hep3B-derived spheres with Sorafenib or SB431542 decreased the expression of stemness markers and *CD73* and *PD-L1*, while the same treatment of PLC/PRF/5-derived spheres showed downregulation of stemness markers *CD44* and *BMI* with elevated expression of *KLF-4*, *CD73* and *PD-L1*. These differences observed may be attributed to cell-line-specific effects, and heterogeneity noticed among cell lines is a feature commonly recapitulated in HCC patients. Importantly, treatment with Sorafenib causes cell death and the residual CSCs can be targeted with immune checkpoint inhibitors as CSCs still have expression of PD-L1, CD73 and CSC markers, regardless of overexpression or downregulation. Furthermore, it is plausible that these treatments as monotherapies may not be effective in eliminating CSCs, and the application of combination therapies, including the immune checkpoint inhibitors, is essential for targeting these CSCs.

In HCC, a study demonstrated that targeting tumour necrosis factor-α (TNF-*α*) can overcome Sorafenib resistance [92]. Another study reported the enrichment of PD-L1 in breast cancer CSCs through TGF-*β*-driven EMT [93]. In addition, the study also suggested that more PD-L1 expression is enriched by EMT in CSCs compared to non-CSCs [93]. However, there are very limited studies investigating the role of EMT, immune checkpoint molecules and therapy resistance in HCC. The limitation of this study is the application of established human HCC cell lines for the enrichment of CSCs that may not be conducive for long-term expansion in culture. Using HCC patient tumour-derived primary cells for spheroid culture may closely recapitulate the status of the primary tumour. Furthermore, organoid cultures from fresh HCC patient tissue samples have been demonstrated to be better preclinical models for propagating 3D spheres with CSC traits, especially in long-term cultures for up to 5 months [94]. As organoid cultures are promising disease models for testing drug efficacy in vitro, the effectiveness of combination therapy for targeting CSC can be tested in this system before moving to animal models. Further studies in animal models to validate the findings from this study are warranted.

## 5. Conclusions

The combination treatment approach with the blockade of EMT and immune checkpoint molecules along with Sorafenib can be an effective treatment modality to target an aggressive and chemoresistant HCC-derived CSC subpopulation. However, further validation of this novel combination approach in in vivo animal model is warranted.

## Figures and Tables

**Table 1:** List of primers for qRT-PCR.

Primers	Sequence (5′-3′)
*CD133* forward	GCTTCAGGAGTTTCATGTTGG
*CD133* reverse	GGGGAATGCCTACATCTGG
*CD44* forward	CCAGAAGGAACAGTGGTTTGGC
*CD44* reverse	ACTGTCCTCTGGGCTTGGTGTT
*KL-F4* forward	CATCTCAAGGCACACCTGCGAA
*KLF-4* reverse	TCGGTCGCATTTTTGGCACTGG
*OCT-4* forward	TTGTGCCAGGGTTTTTGG
*OCT-4* reverse	ACTTCACCTTCCCTCCAACC
*SOX-2* forward	ATGGGTTCGGTGGTCAAGT
*SOX-2* reverse	GGAGGAAGAGGTAACCACAGG
*NANOG* forward	CTCCAACATCCTGAACCTCAGC
*NANOG* reverse	CGTCACACCATTGCTATTCTTCG
*Snai1* forward	GCTGCAGGACTCTAATCCAGA
*Snai1* reverse	ATCTCCGGAGGTGGGATG
TGF-*β*1 forward	TACCTGAACCCGTGTTGCTCTC
TGF-*β*1 reverse	GTTGCTGAGGTATCGCCAGGAA

**Table 2:** List of antibodies.

Antibodies	Cat. No.	Manufacturer	Antibody Category	Dilution
CD133	ab19898	abcam	Primary	1:500
CD44	ab157101	Abcam	Primary	1:500
TGF-*β*1	MA5-15065	Thermofisher	Primary	1:1000
p-Smad 2/3	SC-11769-R	Santa Cruz	Primary	1:100
Smad 2/3	SC-133098	Santa Cruz	Primary	1:100
β-Actin	4967s	Cell Signaling	Primary	1:2000

## Data Availability

The data presented in this study are available on request from the corresponding author.

## Supplementary Materials

The following are available online at https://www.mdpi.com/article/10.3390/curroncol28030200/s1, Figure S1: Silencing of CD73 in human HCC-derived spheres, Figure S2: Hep3B derived spheres treatment with Sorafenib, Figure S3: PLC/PRF/5 derived spheres treatment with Sorafenib, Figure S4: Hep3B derived spheres treatment with SB431542, Figure S5: PLC/PRF/5 derived spheres treatment with SB431542, Figure S6: Silencing of PD-L1 and CD73 in Hep3B derived spheres, Figure S7: Silencing of PD-L1 and CD73 in PLC/PRF/5 derived spheres, Figure S8–S12: Uncropped Western Blot figures.
