# Supplementary material for: Dual Targeting of Sorafenib-Resistant HCC-Derived Cancer Stem Cells
Source: Curr Oncol. 2021 Jun 11;28(3):2150–72. doi: 10.3390/curroncol28030200 (PMC8293268; doi:10.3390/curroncol28030200)
Supplement: Supplementary file 1 [file curroncol-28-00200-s001.zip › curroncol-1254754-supplementary.pdf]

Supplementary Materials

Dual Targeting of Sorafenib-Resistant HCC-Derived Cancer Stem Cells

Ritu Shrestha, Kim R. Bridle, Lu Cao, Darrell H. G. Crawford and Aparna Jayachandran

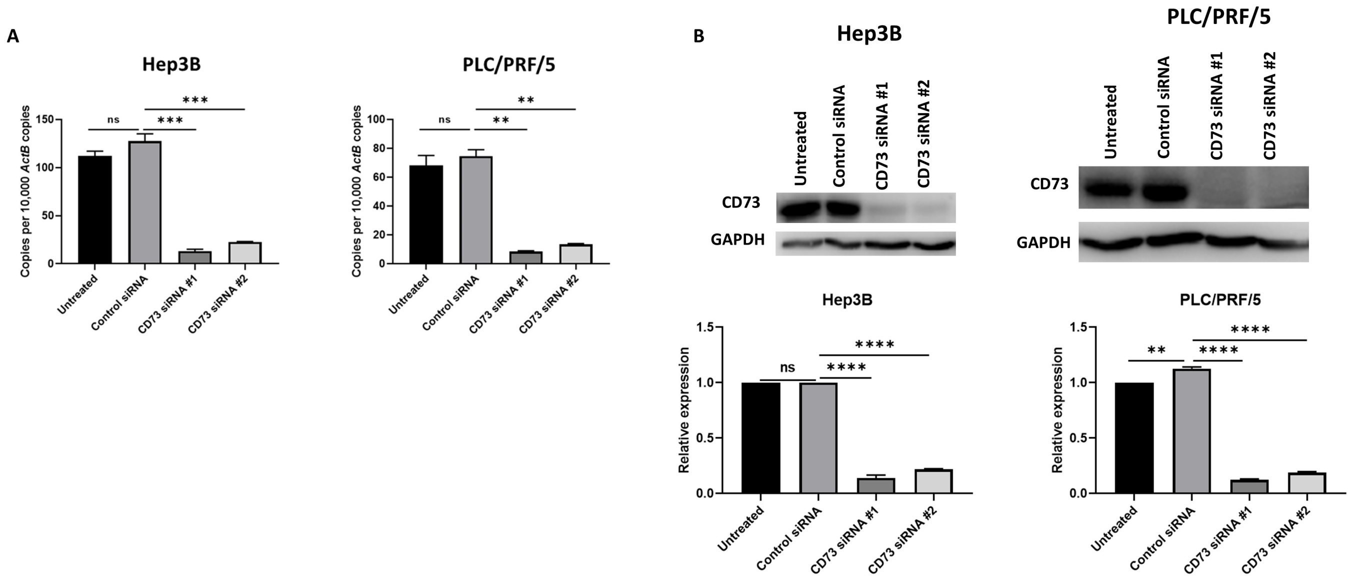

Figure S1. Silencing of CD73 in human HCC derived spheres. Transfection of Hep3B and PLC/PRF/5 cells with two specific CD73 siRNA effectively knockdown CD73 expression as revealed by (**A**) qRT-PCR and (**B**) western blot analysis. GAPDH was used as loading control. ($n = 3$, ** $p < 0.01$, *** $p < 0.005$, **** $p < 0.001$, ns: not significant).

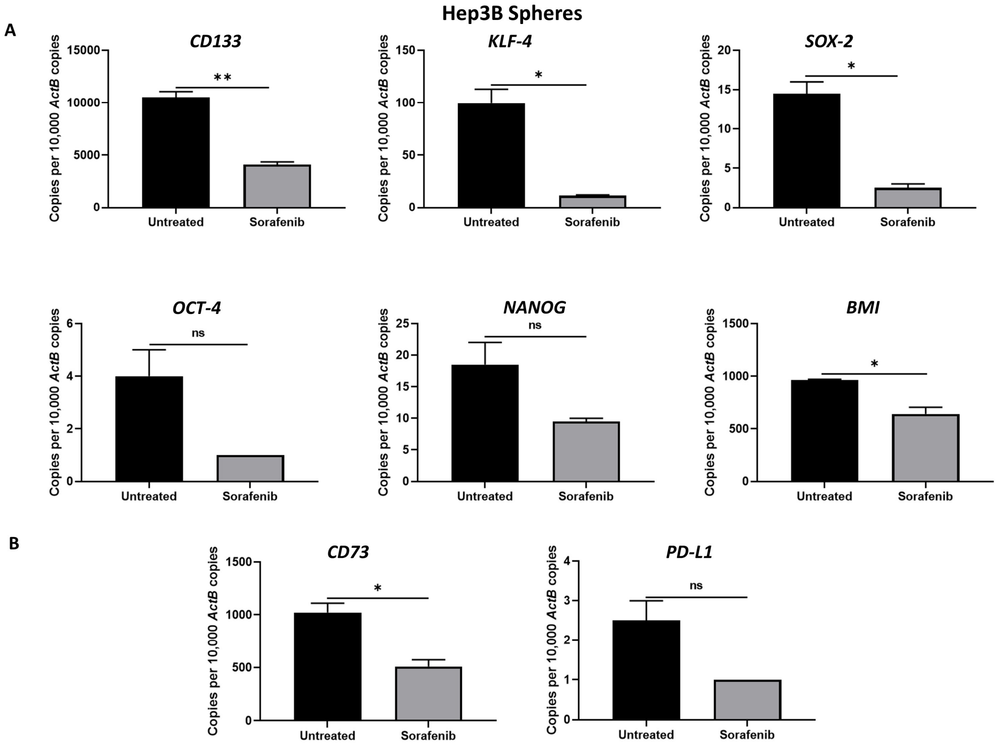

Figure S2. Hep3B derived spheres treatment with Sorafenib. Treatment of Hep3B derived spheres with Sorafenib revealed downregulation of (**A**) stemness markers and (**B**) immune checkpoints by qRT-PCR analysis. ($n = 3$, * $p < 0.05$, ** $p < 0.01$, ns: not significant).

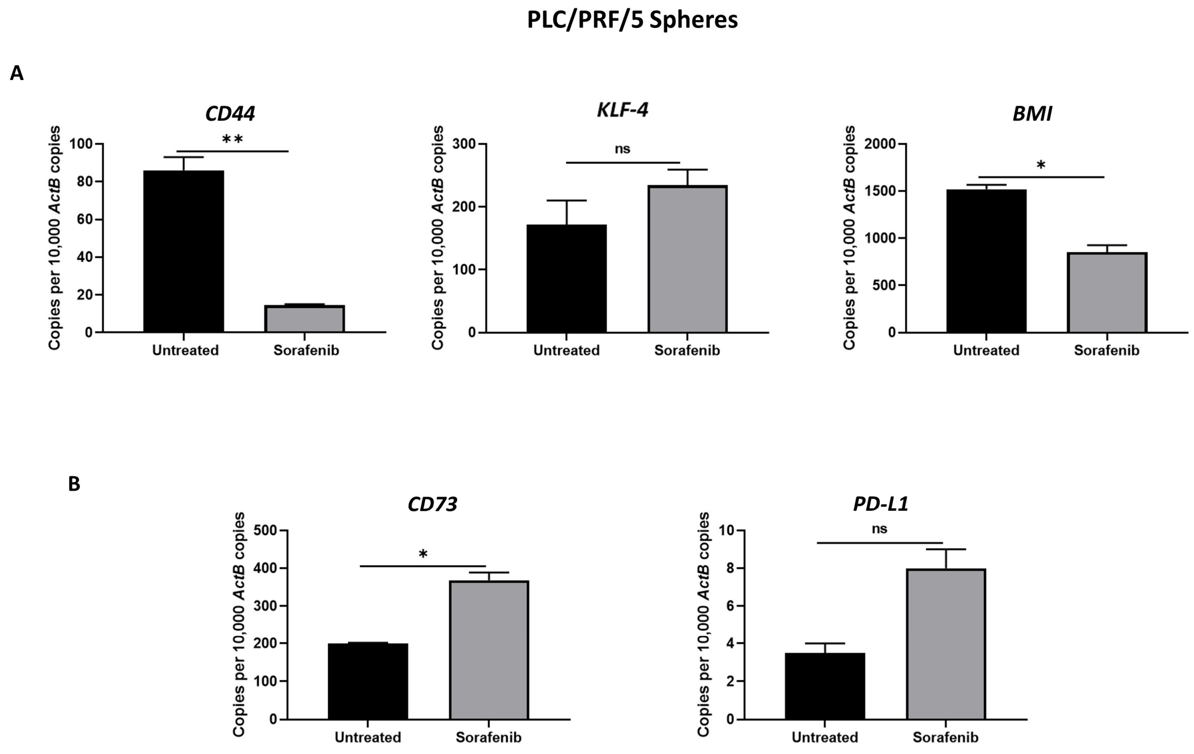

Figure S3. PLC/PRF/5 derived spheres treatment with Sorafenib. Treatment of PLC/PRF/5 derived spheres with Sorafenib revealed downregulation of (**A**) stemness markers *CD44* and *BMI* and upregulation of *KLF-4* and (**B**) upregulation of immune checkpoints by qRT-PCR analysis. ($n = 3$, * $p < 0.05$, ** $p < 0.01$, ns: not significant).

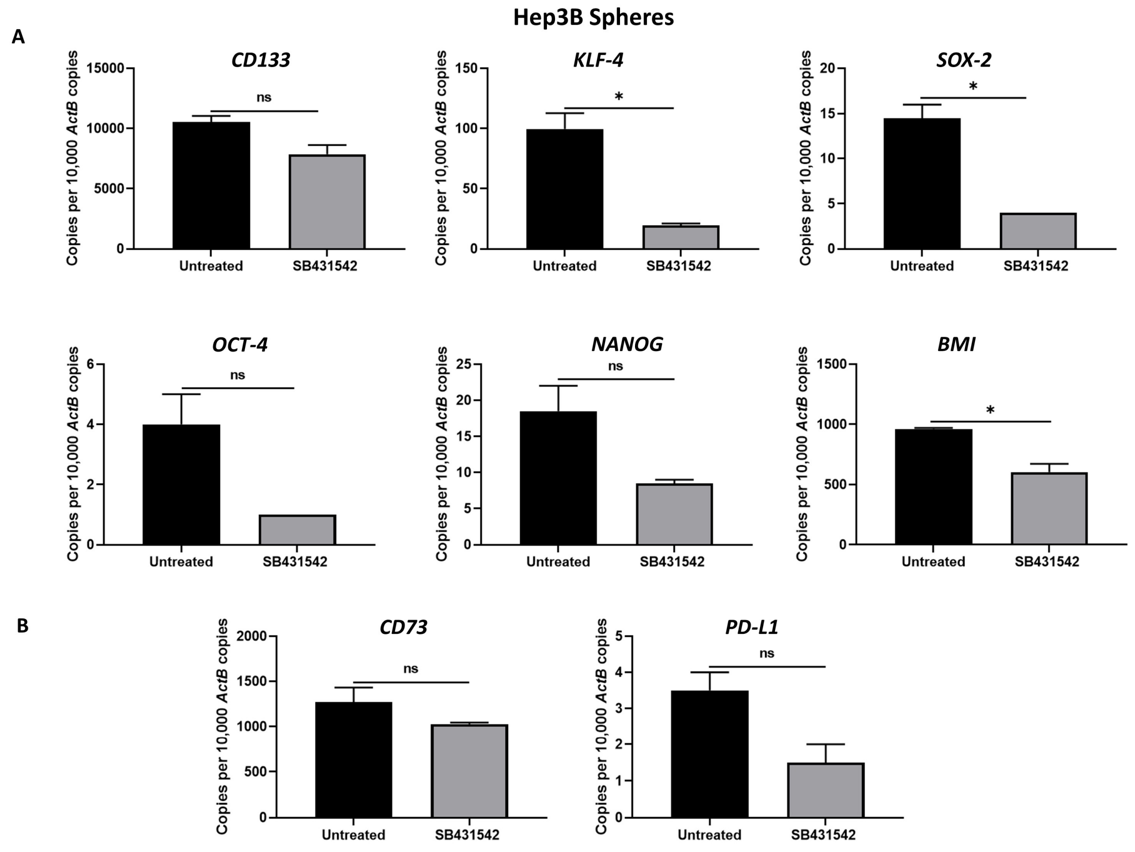

Figure S4. Hep3B derived spheres treatment with SB431542. Treatment of Hep3B derived spheres with SB431542 revealed downregulation of (**A**) stemness markers and (**B**) immune checkpoints by qRT-PCR analysis. ($n = 3$, * $p < 0.05$, ns: not significant).

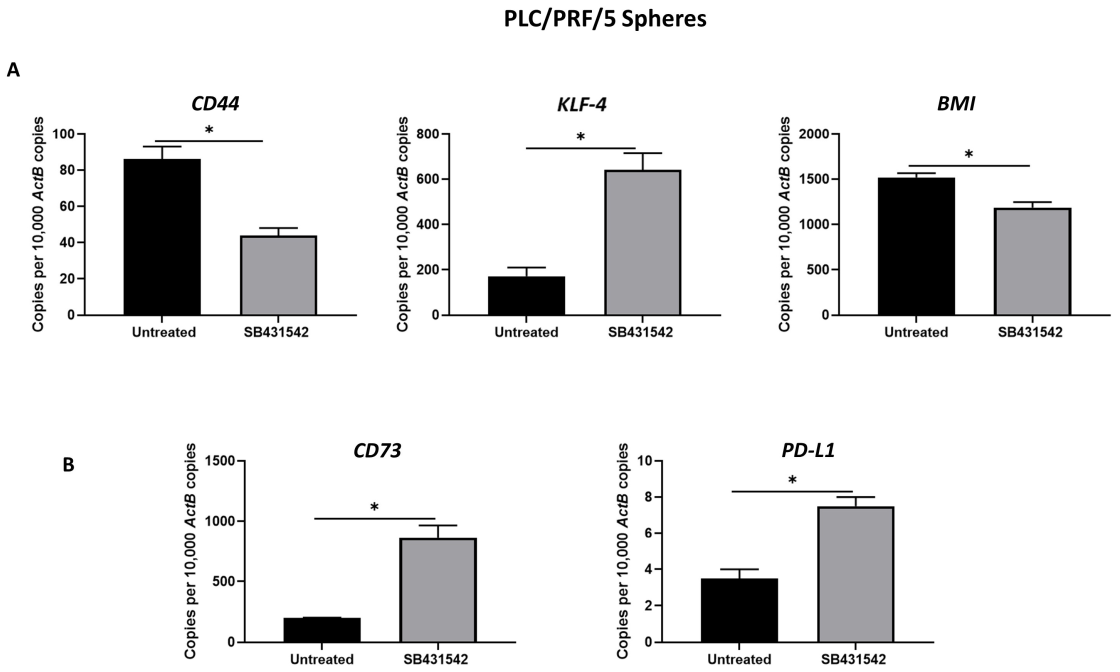

Figure S5. PLC/PRF/5 derived spheres treatment with SB431542. Treatment of PLC/PRF/5 derived spheres with SB431542 revealed downregulation of (**A**) stemness markers *CD44* and *BMI* and upregulation of *KLF-4* and (**B**) upregulation of immune checkpoints by qRT-PCR analysis. ($n = 3$, * $p < 0.05$, ns: not significant).

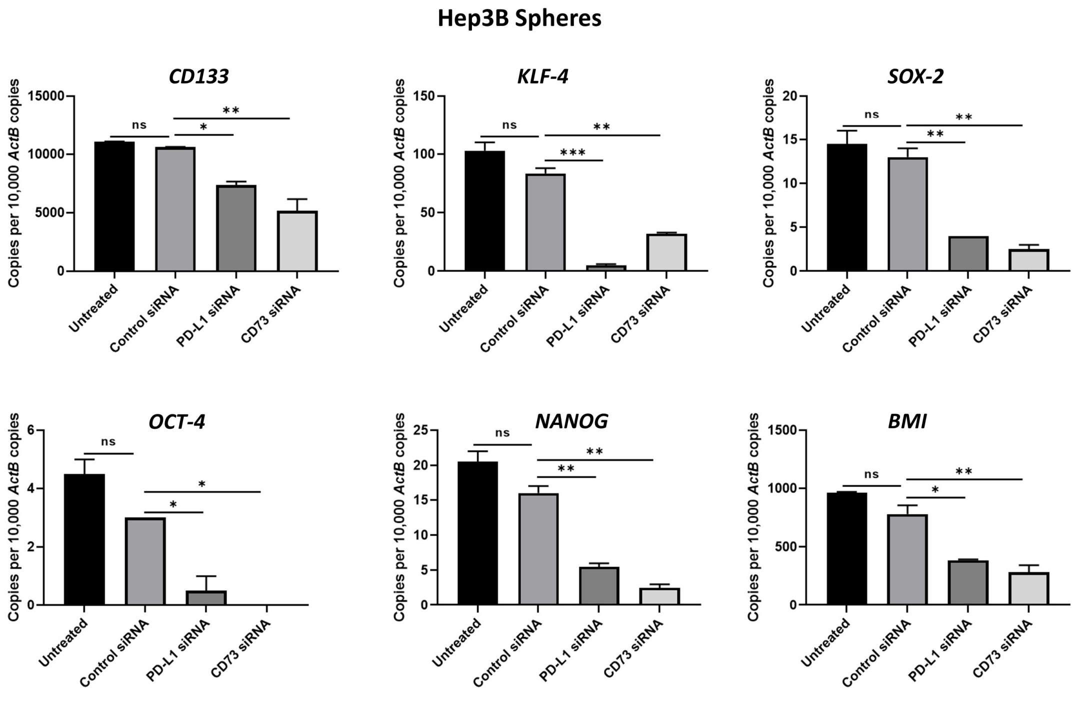

Figure S6. Silencing of PD-L1 and CD73 in Hep3B derived spheres. Transfection of Hep3B cells with PD-L1 or CD73 siRNA decreased the expression of stemness markers as revealed by qRT-PCR analysis. ($n = 3$, * $p < 0.05$, ** $p < 0.01$, *** $p < 0.005$, ns: not significant).

PLC/PRF/5 Spheres

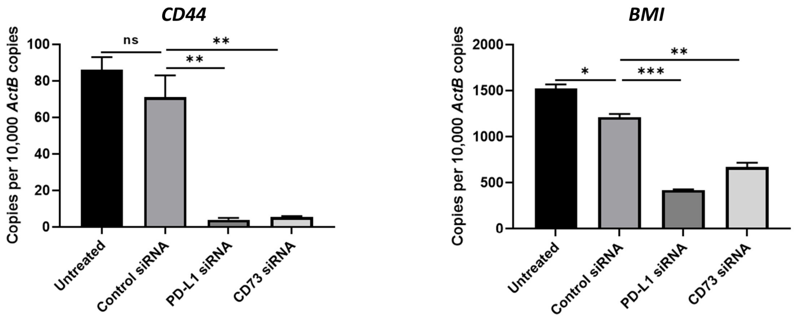

Figure S7. Silencing of PD-L1 and CD73 in PLC/PRF/5 derived spheres. Transfection of PLC/PRF/5 cells with PD-L1 or CD73 siRNA decreased the expression of stemness markers as revealed by qRT-PCR analysis. ($n = 3$, * $p < 0.05$, ** $p < 0.01$, *** $p < 0.005$, ns: not significant).

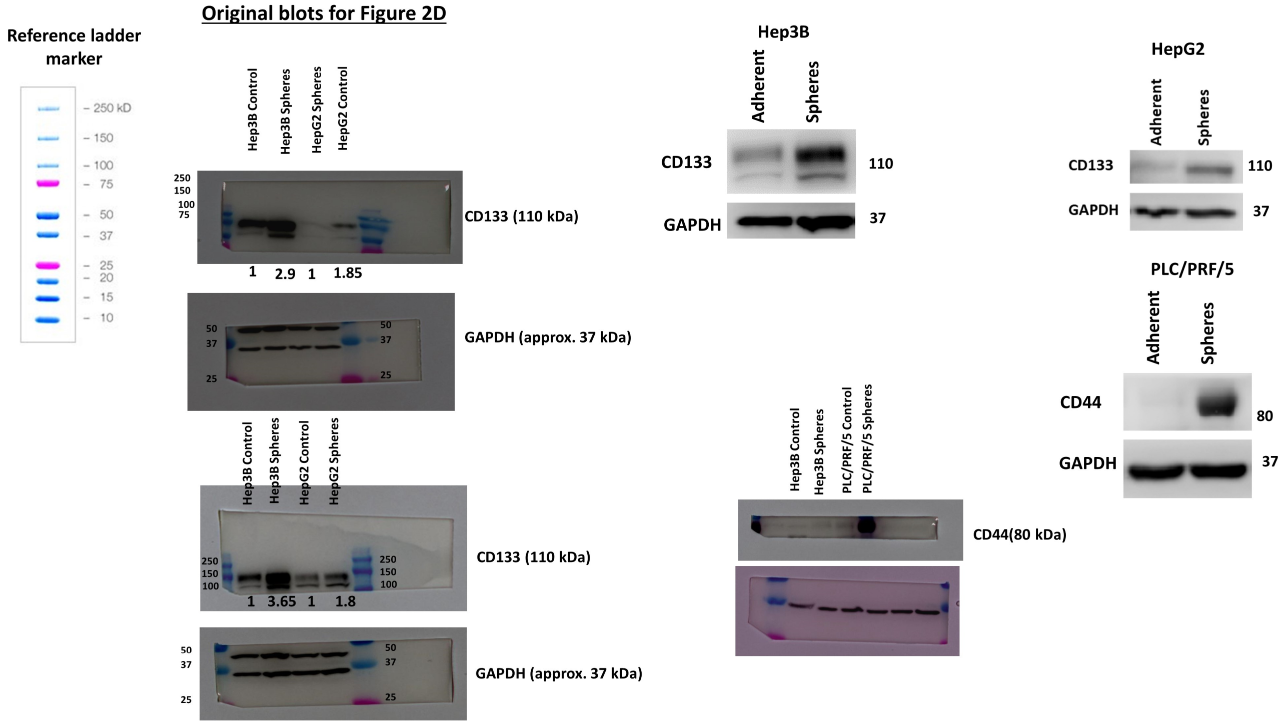

Detail information about Figure 2.

Original blots for Figure 4

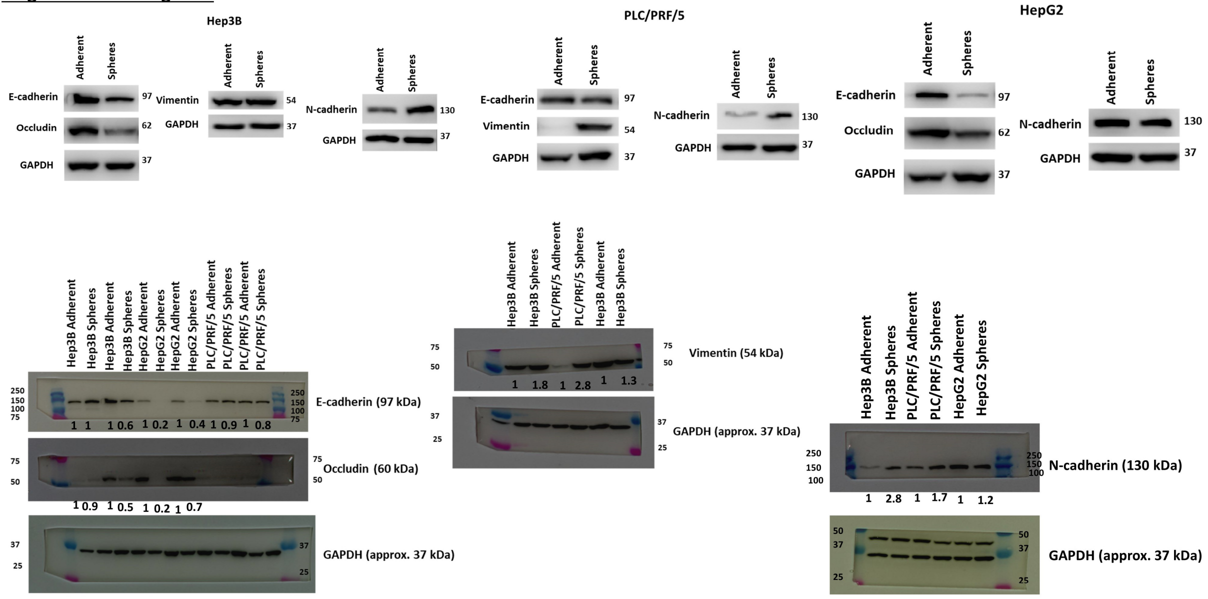

Detail information about Figure 4.

Original blots for Figure 5B

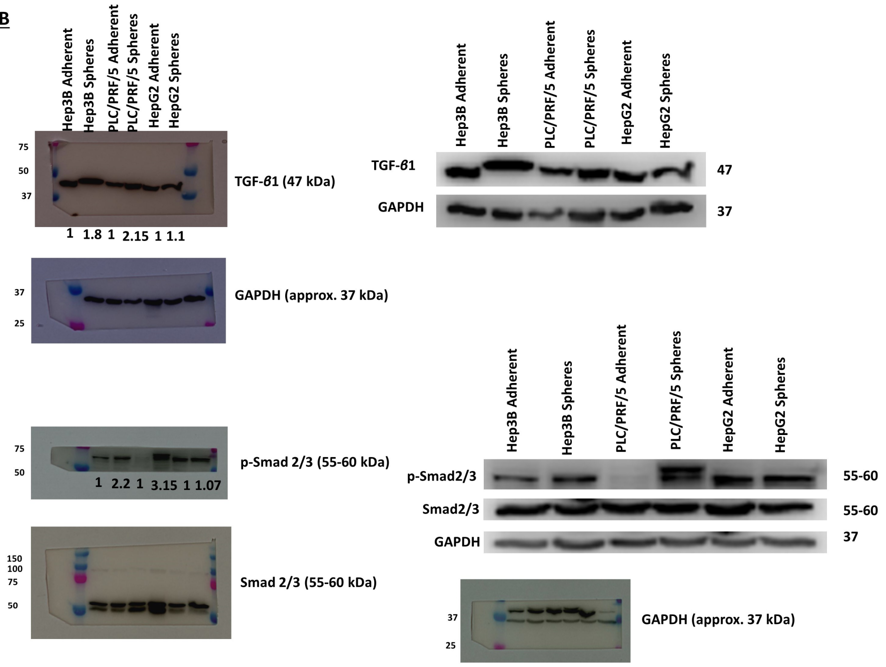

Detail information about Figure 5.

Original blots for Figure 7D

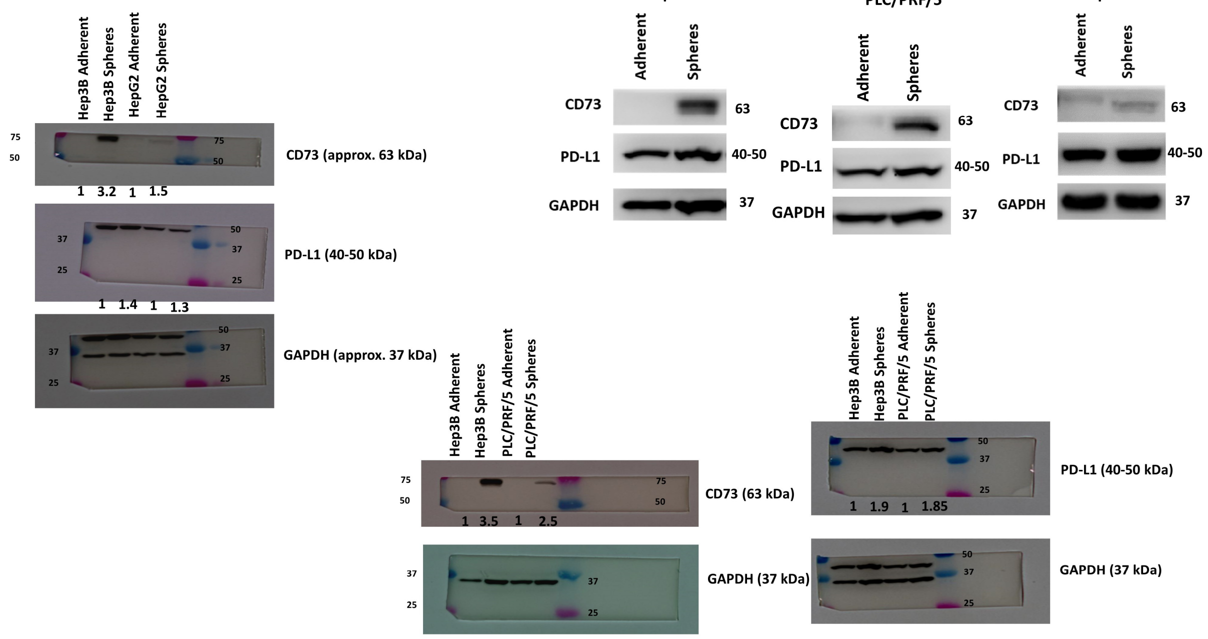

Detail information about Figure 7.

Original blots for Supplementary Figure S1

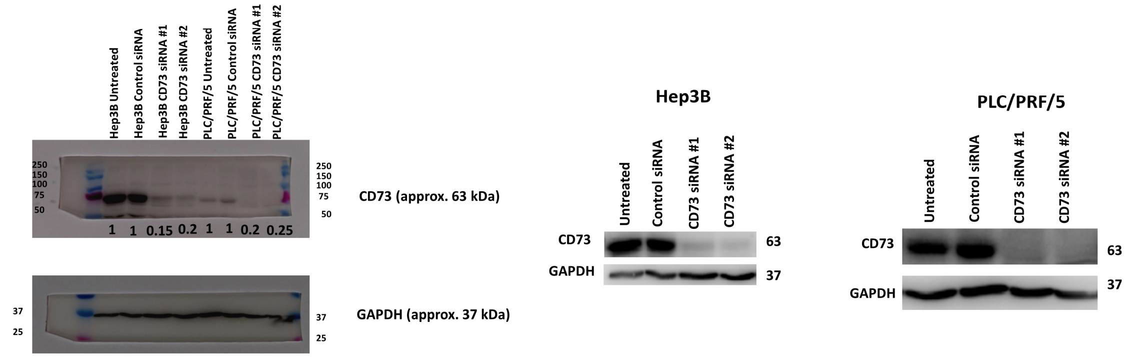

Detail information about Figure S1.
